# The use of the EQ-5D as a measure of health-related quality of life in people with dementia and their carers

**DOI:** 10.1007/s11136-014-0770-0

**Published:** 2014-08-17

**Authors:** Vasiliki Orgeta, Rhiannon Tudor Edwards, Barry Hounsome, Martin Orrell, Bob Woods

**Affiliations:** 1 Division of Psychiatry, University College London, 67-73 Riding House Street, 2nd Floor, Charles Bell House, London, W1W 7EJ UK; 2 Centre for Health Economics and Medicines Evaluation, Bangor University, Bangor, UK; 3 Faculty of Health Sciences, University of Southampton, Southampton, UK; 4 Division of Psychiatry, University College London, London, UK; 5 Dementia Services Development Centre, Bangor University, Bangor, UK

**Keywords:** Health-related quality of life, People with dementia, Agreement, Proxies, Psychometric properties, EQ-5D, Cost-effectiveness

## Abstract

**Purpose:**

To assess the acceptability, validity and inter-rater agreement of self- and family carer proxy ratings of the EQ-5D as a generic health-related quality of life (HRQOL) measure, in people with mild to moderate dementia (PwD) living in the community. A secondary aim was to identify the most important factors influencing self- and family carer proxy ratings of HRQOL, distinguishing between spouse and adult child caregiver ratings.

**Methods:**

Cross-sectional study of 488 dyads using the EQ-5D. Inter-rater agreement was examined using weighted kappa scores, and validity by investigating the association of self- and family carer ratings with clinical variables. Factors affecting HRQOL ratings were analysed using multivariate regression.

**Results:**

The response rate of the EQ-5D was satisfactory; however, agreement between self- and family carer ratings was poor. The most important predictors of PwD and carer ratings of the PwD’s HRQOL were family carer ratings of activities of daily living and mood. Anxiety experienced by the PwD was a significant predictor of self-rated HRQOL, whereas depressive symptoms independently predicted family carer ratings. The type of the caregiving relationship influenced carer ratings of HRQOL, whereby sons and daughters rated HRQOL lower for the PwD compared with spousal caregivers.

**Conclusions:**

People with mild to moderate dementia are able to rate their own HRQOL through a brief generic instrument; however, self-ratings consistently differ from family carer ratings, which should be acknowledged in cost-effectiveness analyses. Spouse caregivers rate HRQOL for the PwD more positively compared to adult children.

## Introduction

Policy makers and health and social care commissioners look to evidence of cost-effectiveness to aid commissioning and priority setting decisions. The National Institute for Health and Care Excellence (NICE) encourages the use of quality-adjusted life years (QALYs) where possible, based on the use of the EQ-5D instrument to generate preference-based health-related quality of life (HRQOL) weightings [1, 2]. Measuring HRQOL is becoming an important objective in dementia research, where the importance of valuing the perspective of the person with dementia is emphasized [3]. The concept of HRQOL refers to the impact of disability on the general well-being of an individual including people with dementia (PwD) [3]. A central component of economic evaluations of health care is assessing the impact of an intervention on HRQOL, using preference-based instruments [1]. These usually consist of two components: a health state classification system and population-based preference weights used to calculate utility scores, weighted using valuation techniques such as standard gamble or time trade-off [4, 5].

Although self-ratings are considered the gold standard in estimating HRQOL in dementia, compromised cognitive function in PwD and varying degrees of capacity to make judgments [6, 7] question the validity of self-ratings. As a result, trials often ask family caregivers or care home workers to offer proxy values instead of or in addition to self-ratings from the PwD. A previous review on the use of EuroQol EQ-5D (EQ-5D) in PwD has suggested that the instrument shows good reliability in comparison with other utility measures for both self- and proxy ratings in people with mild to moderate dementia [3], making it therefore the most common preference-based instrument for cost-utility analysis in this population [3, 8, 9].

Despite, however, the frequent use of preference-based instruments in dementia, the inter-rater agreement between self- and proxy ratings appears relatively low. Past studies examining the possible factors influencing level of agreement [8, 10, 11] have shown that as in several other populations [12, 13], proxies of PwD report higher levels of impairment in functioning [14] and lower levels of well-being [15, 16] in comparison with self-ratings. Theoretical models [17] suggest that the accuracy of a proxy rating may in fact be influenced not only by the type of information evaluated but additionally by characteristics of both the PwD and their carer. In line with this model, proxy ratings of HRQOL are largely influenced by caregiver burden, ability in activities of daily living (ADLs) and levels of depressive symptoms experienced by the PwD [8, 10, 11].

Proxy QOL ratings therefore do not accurately reflect ratings by PwD [18, 19], whereas poor agreement between institutional and family carers [9] poses further concerns for the use of proxy ratings [20]. For example, ratings provided by clinicians have higher construct validity for observable items of the EQ-5D, whereas family carer ratings show higher construct validity for dimensions such as ‘usual activities’ and ‘anxiety/depression’ [8]. When evaluating the different dimensions of HRQOL of the EQ-5D, mobility shows the best agreement, whereas pain presents as the most unstable dimension [9]. Although some studies find that overall agreement is better on ‘observable’ and objective dimensions of the EQ-5D (i.e. ‘mobility’ and ‘self-care’) [8] or higher for people with mild dementia, this is not a consistent finding [9, 19]. General cognition of the PwD influences discrepancies between self- and carer proxy HRQOL ratings, with lower inter-rater agreement when PwD score lower than 10 on the MMSE [9].

There is currently limited evidence on the reliability and validity of the EQ-5D in PwD. So, although several studies support its validity [9], others [19] report issues around self-ratings, such as substantial ceiling effects, indicating that the instrument may not be able to discriminate between comparatively good health states [11]. Little is known about the contribution of the key factors affecting both self- and carer ratings of HRQOL [3] and whether the type of the caregiving relationship that is whether the proxy rater is a spouse or a child of the PwD influences proxy-rated HRQOL. For example, in disease-specific QoL ratings in dementia, spouse proxy ratings are higher compared with child proxy ratings [16, 20].

The purpose of this study was to examine the validity of self- and family carer ratings of HRQOL using the EQ-5D, in a large sample of people with mild to moderate dementia. The main aim was to assess the psychometric properties of the EQ-5D and level of agreement between self- and carer ratings. A secondary objective was to identify predictors of HRQOL by examining the influence of several factors—including characteristics of the PwD and carer, distinguishing between spousal and adult child carer ratings. To our knowledge, the literature to date does not provide any systematic evaluations of differences between spouse and adult child caregivers on perceived HRQOL for PwD. Our hypotheses were that: (1) EQ-5D scores will be higher for people with better function, and fewer depressive and anxiety symptoms, (2) agreement between self- and carer ratings will be stronger for ‘observable’ and objective dimensions of the EQ-5D, and (3) that spousal caregivers will report higher levels of HRQOL for the PwD [16].

## Methods

The sample consisted of 488 people with a diagnosis of dementia according to DSM-IV criteria, along with their carers who had regular contact with the PwD (4 h per week or more) and were assisting the person with basic and instrumental activities. All of the participants were living in the community, in several areas of the UK. The present data were collected as part of the REMCARE study (baseline data), investigating the effects of reminiscence therapy for people with dementia and their family carers [21]. This HTA funded trial was approved by the Multi-Centre Research Ethics Committee in Wales. All people with dementia and their carers gave their consent to participate in the study. The assessment instruments were administered by a team of research assistants.

### Measures

#### EQ-5D

The EQ-5D is a brief generic instrument consisting of a self-administered health index and a visual analogue scale (VAS), a 20-cm scale in which respondents are asked to rate their current health state [22]. It is a brief instrument, representing five dimensions of HRQOL [23], as opposed to QoL in general [24]. The EQ-5D contains five domains: mobility, self-care, pain/discomfort, usual activities and anxiety/depression. There are three levels per dimension: no problems, some problems or extreme problems. For the items measuring experience of pain and anxiety, the three ratings relate to the severity of symptoms. Utility scores quantify HRQOL along a continuum that ranges from −0.59 (worst health) to 1.00 (perfect health). Respondents are asked to mark their current health state on a 100-point VAS scale, with 100 representing the ‘best imaginable health state’ and 0 representing the ‘worst imaginable health state’. People with dementia completed the EQ-5D in an interview format. In the proxy version, carers were asked to answer the questions giving their own view of the person’s QoL, as opposed to attempting to provide the person’s own view.

#### Convergent validity

The following scales were included to examine the validity of self- and carer ratings of HRQOL for PwD.

#### Mood

Depression for people with dementia was measured by the *Cornell Scale for Depression in Dementia* (CSDD) [25]. The CSDD is a 19-item interviewer administered measure, using information from interviewing the PwD and their carer. Anxiety was measured by the *Rating of Anxiety in Dementia Scale* (RAID) [26], which comprises 18 items assessing anxiety, based on a structured interview with the carer and the PwD.

#### Function

The *Bristol Activities of Daily Living Scale* (BADLS) [27] has been developed specifically for use with PwD, as a carer-rated instrument consisting of 20 daily living abilities.

#### Dementia severity

Global severity of dementia was determined using the *Clinical Dementia Rating* scale (CDR) [28], which was administered as a structured interview collecting information in a standard way from both the family carer (informant) and the PwD in relation to memory, orientation, problem solving, community affairs, home and hobbies, and personal care.

#### Caregiver characteristics

Caregivers’ mental health was measured using the *General Health Questionnaire* (GHQ-28) [29], used widely to assess psychological well-being and distress in carers [29, 30]. Stress specific to the caregiving situation was measured by the *Relative’s Stress Scale* (RSS) [31], administered as a self-report measure.

### Statistical analysis

Feasibility was examined by the percentage of missing items and the ability of the EQ-5D to discriminate between health states by observing potential ceiling effects. Agreement between self- and carer ratings was analysed by calculating exact agreement and weighted Kappa coefficients. According to Landis and Koch [32, 33], kappa coefficients between 0.41 and 0.60 indicate moderate agreement, with those below this level reflecting weak agreement. Inter-rater reliability of the utility scores was tested by calculating intra-class correlation coefficients (ICCs), and their confidence intervals, based on one-way ANOVA. Mean utilities between self- and carer ratings were compared by paired *t* tests. EQ-5D data were converted into health utility scores, providing a single evaluation, using the time trade-off method based on the tariff developed for the EQ-5D index UK [33]. To determine the effect of clinical variables on the perception of self- and carer-rated HRQOL utility scores, step-wise multivariate regression analyses were performed. Self-rated HRQOL and carer-rated HRQOL of the PwD were entered as the dependent variables, whereas the predictors were those factors found to be significant in correlational analyses. Separate models were built for spousal versus adult child caregivers. Since several hypotheses were tested per item and score on the EQ-5D, the Bonferroni correction was performed.

## Results

### Sample characteristics

Demographic characteristics of the sample are presented in Table 1. Adult child caregivers had higher levels of education (*t* = −2.20; *p* < 0.05) and were caring for PwD who were older (*t* = −1.03; *p* < 0.05), had greater impairments in BADLS (*t* = −2.28; *p* < 0.001) and lower levels of education (*t* = −3.43; *p* < 0.01). No other differences were observed between adult child and spousal caregivers.

**Table 1:** Demographic characteristics of PwD and their carers

	Overall (*n* = 478) mean (SD) or %	Spousal caregivers (*n* = 344) mean (SD) or %	Adult child caregivers (*n* = 95) Mean (SD) or %
*PwD*			
Age, years	75.5 (7.3)	76.2 (6.8)	82.0 (6.3)
Education, years	15.5 (2.3)	15.8 (3.3)	14.6 (1.2)
Sex			
Female	49.6	36.7	90.6
Marital status			
Married	74.8	100.0	7.6
Widowed	21.1	–	84.9
Divorced	4.1	–	7.5
Living status			
With spouse	68.9	100.0	5.4
With adult children	15.7	–	37.6
Alone	15.4	–	57.0
CDR			
Mild	74.6	75.0	74.1
Moderate	25.4	25.0	25.9
BADLS	15.9 (9.6)	15.6 (9.9)	18.1 (8.4)
CSDD	7.0 (5.0)	6.8 (4.8)	8.0 (5.8)
RAID	8.4 (6.7)	8.4 (6.7)	9.1 (8.6)
*Carers*			
Age, years	69.8 (11.6)	74.0 (7.8)	54.8 (8.8)
Education, years	16.7 (4.8)	16.5 (5.4)	17.7 (2.7)
Sex			
Female	67.1	63.5	79.2
Marital status			
Married	88.4	100.0	66.4
Widowed	3.4	–	4.3
Divorced/single	8.2	–	29.3
Relationship			
Spouse	71.0	100.0	–
Adult child	20.7	–	100.0
Other	8.3	–	–
RSS	21.6 (10.7)	21.9 (10.9)	21.8 (10.1)
GHQ	22.9 (11.8)	23.5 (12.1)	21.7 (10.3)

*Note* *CDR* Clinical Dementia Rating Scale, *CSDD* Cornell Scale for Depression in Dementia, *RAID* Rating of anxiety in dementia, *BADLS* Activities of Daily Living Scale, *RSS* Relative’s Stress Scale, GHQ General Health Questionnaire

### Feasibility and response variability

The response rate for each of the five dimensions was between 97 and 98 %. A total of 95.6 % of the sample responded to all five dimensions. All except 8 participants answered at least 4 of the EQ-5D items, whereas the response rate for the VAS was 98.8 %. There were no differences in EQ-5D utility scores (*t* = −1.03; *p* > 0.05) between people with mild versus moderate dementia. Table 2 shows that ceiling effects were observed for the self-care item, with a total of 80.3 % of PwD responding that they had no problems with self-care. PwD rarely used the ‘extreme problems’ option, with response rates ranging from 0.2 to 4.3 %. Carers used the ‘extreme problems’ option more frequently (range 0–16.8 %). Responses to the visual analogue scale ranged across the full extent of the scales from 0 to 100.

**Table 2:** Distribution of responses of the EQ-5D index scores

Dimension	Category	Overall		Spousal caregiving dyads		Adult child caregiving dyads	
		(*n* = 478)		(*n* = 344)		(*n* = 95)	
		Self	Carer	Self	Carer	Self	Carer
		% of responses	% of responses	% of responses	% of responses	% of responses	% of responses
Mobility	No problems	62.1	49.1	64.7	53.2	52.1	31.3
	Some problems	36.2	48.4	33.5	43.6	46.9	67.7
	Extreme problems	0.2	–	–	–	–	–
	No response	1.4	2.5	1.7	3.2	1.0	1.0
Self-care	No problems	80.5	52.6	78.9	57.5	84.4	34.4
	Some problems	16.1	40.4	17.6	35.5	13.5	59.4
	Extreme problems	1.7	4.6	1.4	3.8	1.0	5.2
	No response	1.7	2.5	2.0	3.2	1.0	1.0
Usual activities	No problems	65.6	23.2	67.6	24.9	60.4	10.4
	Some problems	27.3	57.1	25.7	54.6	32.3	67.7
	Extreme problems	4.3	16.8	4.0	16.8	4.2	20.8
	No response	2.7	2.9	2.6	3.8	3.1	1.0
Pain/discomfort	No problems	53.7	39.8	53.8	41.6	53.1	35.4
	Some problems	40.2	51.6	39.0	49.1	42.7	57.3
	Extreme problems	4.3	6.1	4.9	6.1	3.1	6.3
	No response	1.8	2.5	2.3	3.2	1.0	1.0
Anxiety/depression	No problems	59.8	35.9	60.7	35.3	55.2	35.4
	Some problems	35.9	55.3	34.7	55.8	40.6	55.2
	Extreme problems	2.7	6.4	2.9	5.8	2.1	8.3
	No response	1.6	2.5	1.7	3.2	2.1	1.0

### Agreement between people with dementia and their carers

As can be seen in Table 3, kappa coefficients indicate moderate agreement in the dimension of mobility, which is the most observable of all the dimensions described. Weak agreement was observed for all remaining dimensions, with agreement lower for usual activities across both caregiver groups. Significant but low correlations were observed between the two VAS scores and between ratings of overall HRQOL with ICC concordance weak across spousal and adult child caregivers. As can be seen in Table 4, carers rated their relative’s HRQOL significantly lower.

**Table 3:** Inter-rater reliability of the EQ-5D

EQ-5D	% of exact agreement (*n* = 478)	Kappa-coefficient		
		Overall (*n* = 478)	Self/spouse (*n* = 344)	Self/adult child (*n* = 95)
Mobility	74	0.49	0.51	0.34
Self-care	63	0.25	0.30	0.17
Usual activities	39	0.09	0.09	0.04
Pain/discomfort	58	0.25	0.20	0.38
Anxiety/depression	55	0.20	0.21	0.09
*Intra-class correlation coefficient (95* *% confidence interval)*				
Utility score	–	0.34 (0.28; 0.43)	0.36 (0.26; 0.45)	0.21 (0.13; 0.40)
VAS	–	0.22 (0.13; 0.30)	0.19 (0.10; 0.30)	0.24 (0.11; 0.43)

**Table 4:** Comparisons of EQ-5D utility scores by rater (self, carer, spouse, adult child) and dementia severity

	Overall (*n* = 478)		Spousal caregiving dyads (*n* = 344)		Adult caregiving dyads (*n* = 95)	
	Utility score mean (SD)	Paired-samples *t* test	Utility score mean (SD)	Paired-samples *t* test	Utility score mean (SD)	Paired-samples *t* test
*Overall*						
Self-ratings	0.75 (0.25)	11.84*	0.75 (0.26)	8.65*	0.74 (0.22)	7.35*
Carer ratings	0.59 (0.28)		0.61 (0.29)		0.51 (0.26)	
*Mild dementia*						
Self-ratings	0.79 (0.22)	7.62*	0.79 (0.22)	4.61*	0.79 (0.17)	5.74*
Carer ratings	0.63 (0.27)		0.67 (0.26)		0.53 (0.26)	
*Moderate dementia*						
Self-ratings	0.72 (0.23)	5.41*	0.73 (0.22)	4.88*	0.68 (0.27)	1.91
Carer ratings	0.52 (0.27)		0.52 (0.29)		0.50 (0.26)	

*Note* * *p* < 0.001

### Demographic factors associated with self-rated and carer-rated EQ-5D utility and VAS Scores

Self-ratings of overall physical health (VAS) were associated with dementia severity, *F*(1, 439) = 6.69, *p* = .010, with people with mild dementia perceiving their physical health better overall, than people with moderate dementia. Carer ratings of HRQOL were also associated with dementia severity *F*(1, 439) = 8.65, *p* = .004, with PwD scoring a CDR of 1, perceived by carers to have higher HRQOL. VAS scores as rated by carers were higher for younger PwD (*r* = −0.16, *p* < 0.001). Son/daughter caregivers’ scores were significantly lower than those of spousal caregivers, on both the EQ-5D index, *F*(2, 481) = 4.38, *p* = .013, and VAS scores, *F*(2, 481) = 4.45, *p* = .012. There were no differences in EQ-5D index, *F*(1, 425) = 0.51, *p* = .822, and VAS, *F*(1, 433) = 0.73, *p* = .394, on self-rated HRQOL between spouse and adult child caregiving dyads.

### Multivariate linear regression analysis predicting EQ-5D Index and VAS Scores

Multivariate linear regression analysis (Table 5) showed that, after controlling for all demographic and clinical factors, levels of anxiety and BADLS were independently contributing to self-ratings on the EQ-5D, *F*(4, 352) = 11.12, *p* < 0.001. Activities of daily living were the only significant predictor of self-ratings of health as measured by VAS, *F*(5, 167) = 4.70, *p* < 0.001. Regression analysis showed that carer ratings of overall HRQOL were predicted by depression scores on the CSDD and BADLS, *F*(7, 157) = 21.65, *p* < 0.001. Ratings on the VAS by carers showed that BADLS was the only significant predictor, *F*(6, 343) = 15.35, *p* < 0.001 (Table 5). In spousal caregiving dyads (Table 6), BADLS was the only significant predictor of both self-, *F*(5, 115) = 3.09, *p* < 0.001, carer EQ-5D utility scores, *F*(6, 106) = 19.99, *p* < 0.001, and carer VAS ratings, *F*(7, 106) = 8.71, *p* < 0.001. Model fit for self-rated VAS scores was *F*(5, 113) = 2.41, *p* < 0.05. In adult child caregiving dyads, anxiety measured by the RAID was a significant predictor of self-ratings on the EQ-5D, *F*(5, 32) = 4.32, *p* < 0.01. CSDD scores and BADLS made an independent contribution in predicting both carer-rated EQ-5D utility, *F*(6, 31) = 5.99, *p* < 0.01, and VAS scores, *F*(6, 31) = 5.91, *p* < 0.01, in adult child caregivers.

**Table 5:** Multivariate linear regression analyses for self- and carer ratings of the EQ-5D index and VAS scores

	Utility score				VAS			
	*B*	SE *B*	*β*	*R* ^2^	*B*	SE *B*	*β*	*R* ^2^
*Self-Ratings*								
				.113				.127
CDR	–	–	–		−0.983	.541	.002	
CSDD	−0.303	.004	−.065		−0.397	.427	−.105	
RAID	−0.430	.003	−.168*		−0.473	.470	−.178	
BADLs	−0.690	.002	−.257**		−0.425	.183	−.234*	
RSS	−0.243	.002	.100		0.181	.176	.105	
*Carer ratings*								
				.503				.215
Age of Carer	0.445	.001	.018		0.972	.084	.057	
CDR	0.117	.040	.019		–	–	–	
CSDD	−0.132	.005	−.230*		−0.487	.278	−.128	
RAID	0.152	.003	.004		−0.141	.195	−.050	
BADLs	−0.132	.002	−.463**		−0.626	.116	−.312**	
RSS	−0.453	.002	−.172		−0.752	.131	.042	
GHQ	−0.250	.002	−.010		−0.129	.101	.080	

*Note.* n = 478; CDR, Clinical Dementia Rating Scale; CSDD, Cornell Scale for Depression in Dementia; RAID, Rating of Anxiety in Dementia; BADLS, Activities of Daily Living Scale; RSS, Relative’s Stress Scale; GHQ, General Health Questionnaire; *B* = unstandardized coefficient, SE *B* = standard error of *B;* *β* = standardized coefficient

* p < 0.5, ** *p* < 0.01

**Table 6:** Multivariate linear regression analyses of the EQ-5D utility and VAS scores for spousal and adult child caregiving dyads

	Spousal caregiving dyads (n = 344)								Adult Child caregiving dyads (n = 95)							
	Utility score				*VAS*				Utility score				*VAS*			
	*B*	SE *B*	*β*	*R* ^2^	*B*	SE *B*	*β*	*R* ^2^	*B*	SE *B*	*β*	*R* ^2^	*B*	SE *B*	*β*	*R* ^2^
*Self-Ratings*																
				.123				.100				.445				.251
CDR	0.394	.052	.052		−0.376	.455	−.090		−0.243	.004	−.105		0.608	.621	.165	
CSDD	−0.593	.007	−.129		−0.964	.632	−.246		−0.413	.007	−.111		0.442	.754	.014	
RAID	−0.333	.005	−.099		−0.862	.434	−.029		−0.102	.005	−.471*		−0.902	.486	−.447	
BADLs	−0.613	.003	−.301*		−0.235	.251	−.135		−0.243	.004	−.105		0.733	.454	−.250	
RSS	0.264	.003	.133		0.181	.238	.107		−0.858	.004	−.430		−0.156	.396	−.309	
*Carer ratings*																
				.545				.381				.590				.371
Age of PwD	–	–	–		0.945	.239	.033		–	–	–		–	–	–	
CDR	−0.423	.052	−.050		0.855	.453	.019		0.686	.097	.103		0.959	.709	−.230	
CSDD	−0.112	.007	−.190		−0.535	.602	−.124		−0.212	.008	−.415*		−0.123	.609	−.366*	
RAID	0.129	.005	.030		−0.185	.404	−.059		−0.273	.005	−.094		0.212	.393	.104	
BADLs	−0.133	.003	−477**		−0.654	.258	−.331*		−0.142	.005	−.451**		0.142	.357	−.515**	
RSS	−0.283	.003	−.096		−0.389	.262	−.208		−0.433	.006	−.162		−0.521	.427	−.270	
GHQ	−0.363	.002	−.178		−0.842	.190	−.047		−0.686	.004	−.276		0.525	.311	.293	

*Note* *CDR* Clinical Dementia Rating Scale,* CSDD* Cornell Scale for Depression in Dementia,* RAID* Rating of anxiety in dementia,* BADLS* Activities of Daily Living Scale,* RSS* Relative's Stress Scale,* GHQ* General Health Questionnaire,* B* = unstandardized coefficient, SE* B* = standard error of B,* β* = standardized coefficient

* p < 0.5, ** p < 0.01

## Discussion

### Feasibility of the EQ-5D

The results of our study show that people with mild to moderate dementia are able to respond to and rate their own HRQOL using the EQ-5D. We found that carer ratings were associated with ADLs scores and measures of depression and anxiety, adding to construct validity; however, they were weakly associated overall with self-ratings of HRQOL. Despite demonstrating that people with mild and moderate dementia can rate their HRQOL using the EQ-5D in the context of an interview, we observed a large ceiling effect for the self-care dimension. Although the ceiling effect in EQ-5D is seen when respondents classify themselves as having no problem on any of the five dimensions [34, 35], in the present study ceiling effects were more evident for the dimension of self-care than for other dimensions. This finding is in line with previous research showing that ceiling effects arise even when best health state is still associated with substantial impairments in HRQOL [36].

We found little use by PwD of the ‘extreme problems’ response option in HRQOL, leading to each dimension effectively being a dichotomous scale, which may limit the usefulness of the instrument as an outcome measure in clinical trials of interventions to support PwD. Future studies should examine whether increasing the number of dimensions of the EQ-5D improves response variability, such as comparing the EQ-5D-5L with the EQ-5D-3L. Overall, our response rate for each of the five dimensions was higher in comparison with previous studies [9], possibly due to the fact that most of the sample in the present study had mild dementia.

### Validity of the EQ-5D

When considering agreement between self- and carer ratings on the EQ-5D, the validity of the instrument is poor; however, validity increases when considering the association of the instrument with ratings of mood and function of the PwD. Contrary to previous studies, we did not find any differences in ratings on the basis of gender [9], and dementia severity did not independently predict HRQOL after adjusting for mood and ADLs in the regression analyses. Although our results show that the consistently significant associations between the PwDs’ and carers’ HRQOL ratings and the PwD’s level of functioning provide partial support for the validity of the EQ-5D, overall carer ratings are influenced by factors other than the PwDs’ functioning.

Similar to other populations [37], and non-cognitively impaired healthy older adults [38], mean scores for PwD were higher than mean scores of caregivers, with discrepancies particularly noticeable for the dimension ‘usual activities’. This could be associated with changes in expectations and goals within the context of experiencing a chronic illness [39, 40]. Our finding of significant differences between self- and proxy ratings is consistent with self- versus carer comparisons in previous studies that use the EQ-5D [9, 10, 18] as well as disease-specific QoL scales [15, 16, 41]. For the EQ-5D items, mobility had the best agreement, whereas the least agreement was observed for usual activities, and for the experience of anxiety and depression.

### Factors influencing self- and carer ratings on the EQ-5D and differences in spouse and adult child caregiving dyads

An important contribution of the present study is the observation that the type of the caregiving relationship influenced ratings of HRQOL by carers, whereby sons and daughters rated HRQOL lower for the PwD compared to spousal caregivers. Regression analyses showed that ADLs and depression experienced by the PwD were independent predictors of carer-rated HRQOL, after controlling for caregiver strain, across both types of caregiving relationship. However, in spousal caregiving dyads, ADLs made a contribution in explaining both self-rated and carer-rated HRQOL. In contrast, anxiety in adult child caregiving dyads was contributing most in explaining self-rated HRQOL, whereas higher depression in the PwD and greater impairment in ADLs were significant predictors of carer-rated HRQOL.

Our study shows that when using the EQ-5D, PwD and their carers do not agree in their ratings of the PwD’s quality of life and that carers’ ratings are influenced by type of caregiving relationship with the PwD. These findings therefore question the validity of the instrument, and how well carers’ ratings reflect the PwDs’ view of their quality of life, as there are important differences between self- and proxy ratings. The number of missing responses was small for both self- and carer ratings but important differences between the two ratings indicate that these should be considered in the context of interpretation of quality of life scores and in economic evaluations. Future research should investigate the responsiveness of the EQ-5D in a longitudinal setting and investigate further differences between spousal versus adult child caregivers.

## Limitations

It is important to acknowledge the limitations of our study. Our sample includes only people with mild to moderate dementia living in the community and is therefore not representative of people with dementia in residential care, or those experiencing severe dementia. Cognitive function was not directly measured, so we were not able to evaluate the association between preference scores and cognition. Present findings in relation to carer ratings of HRQOL of PwD may not be generalizable to all carers, as all of the participants interviewed were family carers and were not paid for their provision of care to PwD. A further limitation relates to the potential bias related to self- versus interviewer administration, as previous studies report that this may influence ratings of HRQOL [42].

## Conclusion

Our study extends previous knowledge and sparse literature on the feasibility, reliability and validity of the EQ-5D in assessing HRQOL in PwD. We found significant differences between self-rated and carer-rated HRQOL, indicating that both self- and carer utility estimates should be used in economic evaluations of treatments for PwD and that these are not interchangeable. Further work is needed to validate the application of QALYs in this population. Our results show significant differences between self-rated and carer-rated EQ-5D and VAS scores, and between spouse and adult child caregivers, which raise important questions about the appropriate source of HRQOL information for economic analyses.
